# Lessons from the COVID-19 pandemic: Perspectives of medical students

**DOI:** 10.12669/pjms.37.5.4177

**Published:** 2021

**Authors:** Nadia Saeed, Nismat Javed

**Affiliations:** 1 Nadia Saeed, FCPS (Medicine) Associate Professor, Department of Medicine, Shifa College of Medicine, Shifa Tameer-e-Millat University, Islamabad, Pakistan; 2 Nismat Javed, Final year Medical Student, Shifa College of Medicine, Shifa Tameer-e-Millat University, Islamabad, Pakistan

**Keywords:** Anxiety, COVID-19, Depression, Lifestyle, Medical students, Pandemic

## Abstract

**Objective:**

The objective of the study was to assess the impacts of the COVID-19 pandemic on the mental health and lifestyle of our medical students.

**Methods:**

This observational study was conducted on medical students of Shifa College of Medicine, Islamabad from June to August 2020. The GAD-7 and PHQ-9 questionnaires were used for anxiety and depression assessment. Different aspects of changes in lifestyle were evaluated and students were inquired about their views regarding the COVID-19 pandemic. The chi-square test was applied to assess the associations between levels of anxiety and depression with student’s responses to the concerns and lifestyle changes. The binomial logistic analysis was used to highlight important predictors of anxiety and depression. The Wilcoxon signed-rank test was used to compare the time spent on various activities before and during the pandemic.

**Results:**

There were 234 participants in the study. The depression and anxiety were detected in 151 (64.5%) and 146 (66.7%) students. The college closure led to a significant increase in sleeping time, sedentary time, and time on gadgets (Z=-4.67, Z=-7.23, Z= -6.72, respectively) on the Wilcoxon signed-rank test. The binomial regression analysis identified study years be the significant predictors for the development of depression and anxiety (p<0.05).

**Conclusions:**

Our study emphasizes prioritizing both the physical and mental health of medical students is vital to avoid complications related to the pandemic.

## INTRODUCTION

The psychological impacts of the COVID-19 pandemic have been studied widely especially in medical students who were already found to be more prone to psychological stresses as compared to the general population.^1^-^3^ During the pandemic, several factors were found to have increased the vulnerability of medical students towards anxiety and depression, like closure of the institutes, limitation of clinical competencies and expertise, limitations of the online curriculum delivery and assessment methodologies, and economic and social influences. Also, personal and family health safety concerns while dealing with covid patients along with unfiltered information played a vital role in adding to the medical students’ apprehension.^3^-^8^ Moreover, the pandemic, and its associated lockdown situations have brought significant changes in their lifestyle and behavior including changes in eating patterns, prolonged inactivity, and altered sleeping patterns.^9^

The association of the COVID-19 pandemic with anxiety and depression in medical students is evident from studies. Similarly, the lifestyle changes and physical challenges imposed by the pandemic have been proven in some studies. However, this data seems insufficient from our part of the world as the majority of studies either address the psychological impact or focus on the lifestyle changes. Also, included participants in most of the studies belong to a diverse background of education or occupation. To make a comprehensive care plan for medical students one needs to explore pandemic-related issues in a more exclusive but comprehensive way. Our medical students are the stakeholders of our future health care system. Identification of the pandemic-related impacts is vital to plan remedies and interventions so that our future doctors should stay strong and healthy to face all forthcoming challenges.

Our study highlights these perspectives by providing an overview not only of the psychological impact and its multifactorial associations and predictive factors but also the physical impacts by assessing lifestyle changes. We have also tried to explore upcoming challenges our students foresee and their views over various features of the COVID pandemic.

Our research will help us to identify their needs and requirements especially once they resume regular on-campus activities. This is to ensure that future doctors will not compromise their wellness to battle the mental health front and physical challenges.

## METHODS

The observational study was conducted at Shifa College of Medicine (SCM) from June to August 2020, after approval from the institutional ethics committee (IRB# 177-997-2020, dated June 04, 2020). The inclusion criterion was students pursuing a medical degree. The sample size was calculated using Open-Source Epidemiologic Statistics for Public Health to 235 participants based on an expected population of 600 students, and anticipated frequency of 0.50, 95% confidence intervals, and a design effect of 1.0.^10^ The data was collected using a pre-tested, self-designed, online questionnaire after taking informed consent (questionnaire added as supplementary file). Students who did not provide consent, or had a previously diagnosed mental health disorder were excluded.

The questionnaire contained questions related to demographic data. The psychological impacts were assessed by identifying depression and anxiety. The concept of anxiety was drawn from the DSM-IV definition of generalized anxiety disorder (GAD) and GAD-7 scores were employed for its screening.^11^ Depending on the scores, the anxiety levels were divided into mild (0-5), moderate (6-10), moderately severe (11-14), and severe (15-21). The Patient Health Questionnaire-9 (PHQ-9) was used to detect depression, which contains 9 criteria upon which the diagnosis of DSM-IV depressive disorders is based.^11^ The severity of depression was graded as minimal depression (0-4), mild depression (5-9 points), moderate depression (10 to 14 points), moderately severe depression (15 to 19 points), and severe depression (20 to 27 points). Overall, the PHQ-9 and GAD-7 scores greater than or equal to 10 were considered as cut off for the presence of depression and anxiety, respectively.^12^,^13^

Students, concerns, and opinions related to the COVID-19 pandemic were inquired using the Likert scale with one point implying ‘Strongly Disagree’ and five points implying ‘Strongly Agree’.

The physical impacts were explored by quantifying the changes in their leisure time, sleeping time, study time, exercise time, and time spent on electronic devices. In addition, subjective inquiries about dietary patterns, and body weight changes were made.

To personalize the questionnaire, a section with open-ended questions was presented at the end for commenting on new skills learned or practiced at home, features explored within themselves during the pandemic, the biggest challenge the participants consider in the future, and aspects that participants would remember about the time spent in isolation.

The data collected were analyzed using SPSS version 23. Mean±SD, frequencies, and percentages were calculated for respective variables. The chi-square test was applied to assess the associations between levels of anxiety and depression with student’s responses to the concerns and lifestyle changes. The binomial logistic analysis was used to determine the effect of gender, year of study, and the day scholar or boarder (hostelites) status on the likelihood of having depression or anxiety. The Wilcoxon signed-rank test was used to compare sleeping time, sedentary time, time on gadgets, and exercise time before and during the pandemic. A p-value of <0.05 was considered significant for all statistical tests applied in the study.

## RESULTS

There were 234 participants in the study. Their demographic details are shown in Table-I. The analysis of PHQ-9 questionnaire responses showed mild, moderate, moderately severe, and severe depression in 49 (20.9%), 104 (44.4%), 4 (1.7%), and 43 (18.4%) participants respectively, while 70 (29.9%), 9 (3.8%), and 146 (62.4%) demonstrated mild, moderate, and severe anxiety on the GAD-7 scale. Overall, depression and anxiety were detected in 151 (64.5%) and 146 (66.7%) students.

**Table-I:** Demographic characteristics of participants (n=234).

Characteristics	Results
Mean age (Mean±S.D; years)	20.7 ± 1.5
***Gender***	***Frequency n (%)***
Male	123 (52.6)
Female	111 (47.4)
***Year of study***	
1^st^ year*	46 (19.7)
2^nd^ year*	53 (22.6)
3^rd^ year*	48 (20.5)
4^th^ year**	40 (17.1)
5^th^ year **	47 (20.1)
***Residential status***	
Day Scholars	177 (75.6)
Hostelites	57 (24.4)

* pre-clinical years,

** clinical years

The questions to elicit students’ concerns and opinions about the pandemic indicated that academic loss, future career, friends and family health, and financial crises were the major concerns (Table-II). The chi-square test indicated statistically significant associations (p=<0.01) between the severity levels of depression and anxiety and students’ responses to the pandemic related concerns and life style changes.

**Table-II:** The inquiries about the impacts of COVID-19 pandemic on students’ lives* (n=234).

Inquiries	Participants who agreed or strongly agreed to the inquiries n (%)
I am concerned about my health	229 (97.9)
I am concerned about my family and friend’s health	232 (99.1)
I am concerned about financial crises	230 (98.3)
I am concerned about restricted mobility	226 (96.6)
I am concerned about Social restrictions	224 (95.7)
I am concerned about academic loss	233 (99.6)
I am concerned about Future career	232 (99.1)
I have more leisure time	154 (65.8)
I have more time to sleep	154 (65.8)
I have more time for my gadgets	222 (94.9)
I have more time to study	140 (59.8)
I have more time to spend with my family	224 (95.7)
I have more time for Ibadah	117 (50)

* assessed on likert scales.

The Binomial Regression analysis explained 10% and 66.6% (Nagelkerke R2) of the variance in depression and anxiety and correctly classified 64.5 % and 89.3% cases, respectively. The study year and day scholar/boarder status added significantly to our depression prediction, but gender did not show any significant association. At 95% CI, boarders and preclinical year students were more likely to get depressed (odds ratio: 5.1 and 2.3, respectively). The year of the study added significantly to the anxiety model; the pre-clinical year students were more likely (odds ratio: 56.57) to develop anxiety. The impact of gender and day scholars/boarder remained insignificant.

The Wilcoxon signed-rank test showed that the college closure led to a significant increase in sleeping time, sedentary time, and time on gadgets (Z=-4.67, Z=-7.23, Z=-6.72, respectively) but no significant effect on the exercise time (Z=-0.03, p=0.9) was noted. The mobiles were the most commonly used gadgets (47.7%). On average, participants used 2.4±2.1 hours for academic purposes and 2.1±1.4 hours to seek COVID-19 related information.

When questioned about physical activities, 52 (22.2%) participants were doing yoga, 42 (17.9%) participants were running and 118 (50.4%) participants were not exercising. One hundred and sixty-six students (70.9%) claimed to have changed dietary patterns as 71 (30.3%) participants were eating a larger quantity of meals at a time as compared to pre-pandemic meals, 88 (37.6%) participants were having frequent meals, 52 (22.2%) participants were taking unhealthy meals, and only 7 (2.9%) participants had started healthy meals. The lifestyle changes resulted in weight gain in 56 (23.9%) students, weight loss in 30 (12.8%) students, and 69 (29.5%) had no weight changes. Few students did not respond to the questions about weight changes.

Upon inquiring about new skills acquired or practiced, 13 (5.5%) participants had started cooking, 10 (4.2%) opted for gardening, and 51 (21.8%) were vigilant about the cleanliness of their home, 12 (5 %) attended E-workshops and seminars, whereas 167 (71.4%) participants did not practice anything new. Furthermore, 82 (35%) participants answered that their anxiety was a new attribute, while 17 (7%) claimed that they can be good researchers.

Fifty-one (21.79%) students thought that the professional examination and other academic tasks would be the biggest challenge for them once the on-campus academic activities would resume, whereas 24 (10.25%) participants believed that sustaining the motivation to resume their healthy lifestyle acquired during the COVID-19 pandemic, would be the real challenge in future.

Two hundred and twelve (90.59%) students thought that they would never want to remember these days. Twelve (5%) said that the pandemic would be recalled as the most stressful, worrisome, isolated, and longest painful time of their lives, whereas 5 (2.1%) students would remember it as a relaxed time.

## DISCUSSION

A systemic review estimated the overall mean prevalence of anxiety as 33.62% in Pakistan in 2004.^14^ Even before the pandemic, it was observed in multiple national and international studies that medical students and health care workers have an increased prevalence of anxiety and depression as compared to the general population.^1^,^2^ A study from Karachi found that mild depressive symptoms were present in 20% of medical students and all had some degree of anxiety.^15^ Studies from the twin cities of Islamabad and Rawalpindi detected anxiety in 47.7% of students and depression in 35.1%.^16^ Abrar A et al., observed 39.6% of students had anxiety and depression in SCM in 2014.^17^

After the COVID-19 pandemic, a global rise in the prevalence of anxiety and depression was noticed in medical students. Since isolated national data on medical students is scarce, correlating our findings with these studies might not be judicious, however, our results did show an obvious increase in the prevalence of medical students’ anxiety and depression when compared to the baseline findings from the same institute and are consistent with the outcomes of many international studies.^17^

In Pakistan, a health emergency was announced in mid-March 2020 leading to the immediate closure of academic institutes. At SCM, online academic activities were started the very next day of college closure and were refined subsequently. However, certain feasibility and acceptability issues were observed. Almost all of our students expressed their concerns over the academic loss. Professional examinations were the biggest challenge they foresaw after the resumption of regular academic activities. The sudden college closure and disruption in the timeline for the overall academic calendar, loss of hands-on activities, and online learning system and assessment methodologies might be contributing to this concern, as is identified in several other studies.^3^,^7^

As compared to their selves, students seemed more concerned for the health of the family and friends. Studies have detected 11% to 19% of health science students getting infected with COVID, though data about the prevalence of COVID infection in their relatives is deficient.^3^,^18^ Considering the incidence of more complications and lesser recovery rates, students might be more concerned for their families especially the elderly.^19^

Gender was an insignificant predictor of anxiety and depression, which is contradictory to most of the pre-covid studies, in which female medical students were found to have more anxiety and depression.^1^,^15^,^17^ Few recent studies, also could not relate gender with covid related psychological stress.^3^ Rather few studies found that females had more positive attitudes and better practices towards covid-19 than males.^20^

Before the pandemic, an increased prevalence of psychological stresses was detected in hostelites as compared to day scholar medical students and was linked with adjustment problems.^15^ Studies demonstrated lower anxiety in students who were living with parents during the COVID pandemic.^21^ Our hostelites, were more likely to get depressed, however, this impact was not observed with the prediction of anxiety. Although after campus closure, the hostels were evacuated and students returned to their homes in other cities or countries, but the sudden dislocation to uncertain environments, flight closures, and increased distance from a well-known health care facility could have contributed to this effect.

Our study showed a significant tendency of the pre-clinical group for both depression and anxiety and this relation was stronger for anxiety. Before the pandemic, the psychological stresses were found prevalent in pre-clinical years and then progressively got better in successive years and was attributed to a new environment, increase workload, and demand for medical studies.^17^,^22^ Additional assumptions may be that the clinical-year students were more knowledgeable about COVID, and reliable knowledge helped to reduce anxiety and depression.^23^

Our data provided significant evidences of increase in sleeping and sedentary time along with increased time on gadgets, unhealthy dietary patterns, and the tendency for weight gain. Although we did not quantify some changes in terms of calorie intake and differences in weight, considering the educational status of medical students we expect their interpretation to be reliable. Many studies have highlighted the short and long-term consequences of physical inactivity and unhealthy eating practices during this outbreak.^24^ In addition, the increased prevalence of psychological stress has been linked to a sedentary lifestyle, weight gain, and increased use of gadgets, so both perspectives, physical and mental, have to be addressed to get a better outcome.^23^,^25^,^26^

Considering all the facts and figures, the way forward for our medical students should include indigenous need assessments, identification of prominent stressors, and the guidelines formulation to engage students in productive and healthy activities.^27^ Students should be offered stress management training, physical exercise plans, and proper referral to a psychiatrist, wherever necessary. Provision of a safe and healthy environment of hostels, and hospitals, and preferably a hybrid model for academic sessions with limited but quality time clinical exposures should be considered.

At SCM, online academic activities and assessments were started at the earliest, with a commitment that there should be no time delay for students to graduate. The faculty remained involved in students’ collective and individual counselling and psychiatrist help was taken wherever needed. Academic sessions were repeated multiple times and remedial exams were taken to improve students’ confidence and achieve required competencies.

### Limitations of the study

There were a few strengths and limitations of the study. This study had almost 100% participation rate and strong p-values of the components analyzed. However, it was single centered study and there was a lack of baseline psychological assessment. Our data is self-reported and we did not quantify dietary and weight changes.

## CONCLUSION

This study highlights the increased prevalence of COVID-19 related anxiety and depression in medical students along with tendencies towards unhealthy lifestyles. Risk stratification and intervention must be made for both the physical and mental wellbeing of medical students to meet future challenges.

### Authors’ Contributions:

**NJ** contributed to data acquisition, and its analysis.

**NS** analyzed and interpreted the data and drafted the manuscript.

Both authors revised it critically for important intellectual content and approved the final version to be published. Both authors agree to be accountable for all aspects of the work. Both of the authors made substantial contributions to the conception and designing of the work.
